# Online Health Information–Seeking Among Older Women With Chronic Illness: Analysis of the Women’s Health Initiative

**DOI:** 10.2196/15906

**Published:** 2020-04-09

**Authors:** Mina S Sedrak, Enrique Soto-Perez-De-Celis, Rebecca A Nelson, Jennifer Liu, Molly E Waring, Dorothy S Lane, Electra D Paskett, Rowan T Chlebowski

**Affiliations:** 1 City of Hope National Medical Center Duarte, CA United States; 2 Instituto Nacional de Ciencias Medicas y Nutricion Salvador Zubiran Mexico City Mexico; 3 University of Connecticut Storrs, CT United States; 4 Stony Brook University School of Medicine Stony Brook, NY United States; 5 Ohio State University Comprehensive Cancer Center Columbus, OH United States; 6 The Lundquist Institute Torrance, CA United States

**Keywords:** online health information–seeking, digital health, technology, chronic disease, internet

## Abstract

**Background:**

Understanding how older patients with chronic illnesses use the internet to obtain health information is relevant for the design of digital interventions aimed at improving the health and well-being of adults aged 65 years and older; this cohort represents the sickest, most expensive, and fastest-growing segment of the US population.

**Objective:**

The objective of our study was to describe online health information–seeking behavior among older patients with chronic illnesses and to compare the characteristics of patients who report using the internet to obtain health information with those who do not.

**Methods:**

The study population included 72,806 women aged 65 years and older enrolled in the Women’s Health Initiative (WHI), a national cohort study, who completed a 2014 supplemental questionnaire assessing everyday technology use and internet use for researching health conditions. Comparisons were made between participants with and without a history of chronic illness and between users and nonusers of online sources for health information. Multivariate logistic regression was used to estimate odds ratios (ORs) and 95% CIs.

**Results:**

Of the total, 59% (42,887/72,806) of older women used the internet for health information. Compared with women who did not use the internet to obtain health information, those who used the internet were younger (median age: 76 vs 81 years), more likely to be non-Hispanic white (90% [38,481/42,887] vs 87% [26,017/29,919]), earned a higher income (over $US 50,000: 55% [23,410/42,887] vs 33% [9991/29,919]), achieved a higher educational level (more than high school: 87% [37,493/42,887] vs 75% [22,377/29,919]), and were more likely to live with a partner (52% [22,457/42,887] vs 36% [10,759/29,919]) (all *P*<.001). Women with Alzheimer disease were least likely to report online health information–seeking compared to those without the disease (OR 0.41, 95% CI 0.38-0.43). In contrast, women with a recent diagnosis of cancer, within the previous 2 years (OR 1.23, 95% CI 1.11-1.36) or 2-5 years ago (OR 1.09, 95% CI 1.00-1.19), were most likely to use the internet for health information.

**Conclusions:**

Nearly 6 in 10 older women participating in the WHI reported using the internet to obtain health information. Patients recently diagnosed with cancer are more likely to be looking for health information online, even after adjustment for age, suggesting that these patients may have a greater need for digital health resources.

## Introduction

Digital health technology has been proposed as a potential tool to improve the quality, cost, and safety of health care for older patients [1]. Despite the widespread availability of health information on the internet and recent increases in internet use among older adults in the United States [1,2], there is limited data regarding internet usage among older adults with chronic illnesses for seeking health information [3]. Understanding how older patients with chronic illnesses obtain information is relevant for the design of educational materials and strategies aimed at improving awareness and empowering older adults.

Most of what we know about older adults’ use of technology comes from prior research that did not examine online health information–seeking behavior by the presence of particular health conditions. Research by the Pew Research Center shows that older adults remain largely disconnected from the digital world, with one-third of adults aged ≥65 years never having used the internet, and roughly half (49%) without internet services at home [2]. Moreover, a recently published analysis of the National Health and Aging Trends Study [1] showed that the use of everyday technology in older adults (aged ≥65 years) was below that of the general population. For instance, only 16% of older adults obtained health information using health technology, compared to up to 60% in younger populations. However, most patients in this cohort considered themselves to be in excellent or very good health. Interestingly, older adults with more comorbidities and those taking more medications were more likely to use digital health technologies [1]. In another study that evaluated patients hospitalized with acute coronary syndrome, 31% of which included older adults (aged ≥65 years), 57% of patients looked online for health information, with no difference by history of type 2 diabetes, hyperlipidemia, hypertension, cardiovascular disease, or cancer [3]. There was also no difference by age groups. In addition, few qualitative studies [4,5] have been conducted to explore the challenges patients with multiple chronic conditions face when using technology for health-related purposes. These prior studies have limited generalizability, and it remains unclear whether existing technological tools are meeting the needs of patients with high levels of illness burden.

Our objective was to assess the frequency of online health information–seeking among patients with chronic illnesses compared to patients without chronic conditions and to examine characteristics associated with online health information–seeking.

## Methods

### Study Design, Data Collection, and Study Population

We examined online health information–seeking among a subset of older participants in the Women’s Health Initiative (WHI). The WHI study included 61,808 postmenopausal women, aged 50-79 years, enrolled at 40 US clinical centers between 1993 and 1998. Women participated in randomized clinical trials with three overlapping components (n=68,132) or an observational study (n=93,676) [6,7]. Follow-up of participants is ongoing, and the study was approved by the institutional review boards of all participating institutions. All participants provided written informed consent. Research study staff involved in data collection were trained, certified, and recertified annually to carry out specific data collection procedures.

For this study, we included 72,806 postmenopausal women (representing 92% of all active participants) aged ≥65 years, who participated in the WHI cohort study and completed a 2014 supplemental survey on technology and internet use for researching health conditions. This study examined deidentified data. As the current analysis does not meet the criteria for research on human subjects, it did not require approval from an institutional review board.

### Measures

In 2014, as part of the WHI Extension Study Supplemental Questionnaire (Form 156), participants were asked about their use of mobile phones, other mobile devices, and computers to access the internet (Yes or No). Additionally, they were asked whether they used the internet to search for health information (“Do you use the Internet to look for health information?”; responses were Yes or No). Multimedia Appendix 1 presents a complete list of technology questions included in the WHI survey [8]. Age, race or ethnicity, annual household income, and smoking status, and medical conditions were self-reported at baseline.

### Statistical Analysis

We examined the characteristics of participants who reported using the internet to obtain health information compared to those who did not using chi-square tests for categorical variables and the Wilcoxon signed-rank test for continuous variables. We considered 2-sided *P* values <.05 to be significant. Multivariate logistic regression was used to estimate odds ratios (ORs) and 95% CIs of online health information–seeking among women with and without chronic illness. We performed all analyses using SAS software (SAS Institute Inc).

## Results

Of the total participants, 59% (42,887/72,806) reported using the internet to obtain health information. Compared with women who did not use the internet to obtain health information, those who used the internet were younger (median age: 76 vs 81 years); more likely to be non-Hispanic white (90% [38,481/42,887] vs 87% [26,017/29,919]); earned a higher income (over $US 50,000: 55% [23,410/42,887] vs 33% [9991/29,919]); achieved a higher educational level (more than high school: 87% [37,493/42,887] vs 75% [22,377/29,919]); were more likely to be non-smokers (94% [40,203/42,997] vs 91% [27,108/29,919]); and were more likely to use other technology including mobile phones (93% [39,670/42,887] vs 76% [22,662/29,919]), computers (96% [41,042/42,887] vs 47% [14,097/29,919]), text messaging (47% [20,343/42,887] vs 22% [6442/29,919]), email (94% [40,485/42,887] vs 41% [12,153/29,919]), and smartphones (46% [19,843/42,887] vs 15% [4470/29,919]) (all *P*<.001; Table 1). Women who used the internet to obtain health information were more likely to live with a partner (52% [22,457/42,887] vs 36% [10,759/29,919]) but were less likely to live alone (34% [14,415/42,997] vs 41% [12,383/29,919]) or with others (4% [1890/42,887] vs 6% [1646/29,919]). Internet users were less likely to report frequent utilization of special services (11% [4550/42,887] vs 16% [4863/29,919]), assisted living (2% [715/42,887] vs 5% [1515/29,919]), and nursing home stays within the last year (2% [691/42,887] vs 3% [830/29,919]) (all *P*<.001).

**Table 1:** Characteristics of older women participating in the Women’s Health Initiative in relation to online health information–seeking.

Demographic characteristics		Used the internet for health information (n=42,887)	Did not use the internet for health information (n=29,919)		*P* value
Age at enrollment (years), median (IQR)		59 (55-63)	64 (59-68)		<.001
Age at time of survey (years), median (IQR)		76 (73-80)	81 (76-85)		<.001
**Age group at time of survey (years), n (%)**				<.001	
	65-74 years	17,495 (41)	5461 (18)		
	75-84 years	21,034 (49)	15,633 (52)		
	≥85 years	4358 (10)	8825 (30)		
**Race/ethnicity, n (%)**				<.001	
	Non-Hispanic white	38,481 (90)	26,017 (87)		
**Annual household income (US $), n (%)**					<.001
	<20,000	2228 (5)	4324 (15)		
	20,000-50,000	15,260 (36)	13,898 (47)		
	>50,000	23,410 (55)	9991 (33)		
**Education, n (%)**				<.001	
	High school or less	5103 (12)	7357 (25)		
	More than high school	37,493 (87)	22,377 (75)		
**Living situation, n (%)**					
	Lives with partner	22,457 (52)	10,759 (36)		<.001
	Lives alone	14,415 (34)	12,383 (41)		<.001
	Lives with other (child, relative, etc)	1890 (4)	1646 (6)		<.001
	Receives special services	4550 (11)	4863 (16)		<.001
	Resides in a place with special services	715 (2)	1515 (5)		<.001
	Stayed in a nursing home in the past year	691 (2)	830 (3)		<.001
**Smoking status, n (%)**					<.001
	Nonsmoker	40,203 (94)	27,108 (91)		
	Smoker	746 (2)	723 (2)		
**Media/internet use characteristics, n (%)**					
	Owns a mobile phone	39,670 (93)	22,662 (76)		<.001
	Uses a computer	41,042 (96)	14,097 (47)		<.001
	Receives text messages on a mobile phone	20,343 (47)	6442 (22)		<.001
	Uses email	40,485 (94)	12,153 (41)		<.001
	Uses the internet (for any purpose)	40,934 (95)	10,974 (37)		<.001
	Uses a smartphone	19,843 (46)	4470 (15)		<.001

**Figure 1:**
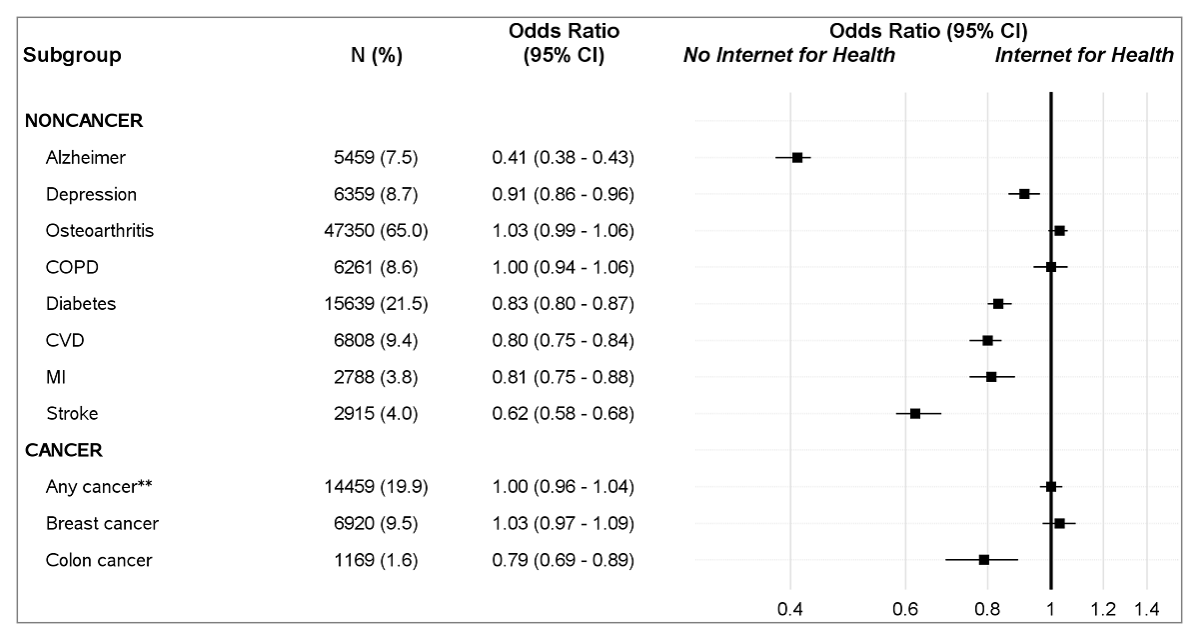
Online health information–seeking behavior in relation to chronic illness status among older women. Diagnosis might have happened at any time prior to the survey. COPD: chronic obstructive pulmonary disease; CVD: cardiovascular disease; MI: myocardial infarction. *All factors were adjusted for the current age group, race, income, and education. **Cancer sites included in the survey: anal, adrenal, appendix, the base of the tongue, biliary, bladder, bone, brain, breast, cerebrospinal, cervical, colon, endocrine, esophagus, eye, gallbladder, genital, gum, hypopharynx, kidney, larynx, leukemia, liver, lung, lymphoma, myeloma, mouth floor, nasopharynx oropharynx, other digestive, ovary, palate, pancreas, parotid, peritoneum, renal pelvis, respiratory, salivary, sinus, stomach, thymus, thyroid, tongue, tonsil, trachea, ureter, urinary, vagina, vulva, and other cancers.

Women with a history of many specific health conditions were less likely to report online health information–seeking (Figure 1). For example, compared to those without a disease, patients with a disease were less likely to use the internet for health information if they had a diagnosis of Alzheimer disease (OR 0.41, 95% CI 0.38-0.43), stroke (OR 0.62, 95% CI 0.58-0.68), colon cancer (OR 0.79, 95% CI 0.69-0.89), cardiovascular disease (OR 0.80, 95% CI 0.75-0.84), myocardial infarction (OR 0.81, 95% CI 0.75-0.88), diabetes (OR 0.83, 95% CI 0.80-0.87), and depression (OR 0.91, 95% CI 0.86-0.96). The only health condition associated with a higher likelihood of online health information–seeking was a recent cancer diagnosis; women diagnosed with cancer within the previous 2 years or 2-5 years were more likely to seek health information online than women without a history of cancer (OR 1.23, 95% CI 1.11-1.36 and OR 1.09, 95% CI 1.00-1.19, respectively). There were no differences in online health information–seeking among those with osteoarthritis, chronic obstructive pulmonary disease, and breast cancer.

## Discussion

### Findings

Nearly 6 in 10 postmenopausal women participating in the WHI reported using the internet to obtain health information. Variables associated with less internet usage for health information were older age, nonwhite race, high school education or less, and an income of US $50,000 or less. Although women with several specific health conditions were less likely to engage in online health information–seeking, those who were diagnosed with cancer in the past 5 years were more likely to look to the internet for health information [9].

There are several important implications of this study. First, a large proportion of older adults are using the internet to seek health information. Our findings add to the existing literature [3] by expanding our understanding of technology use in older adults. A recently published analysis of the National Health and Aging Trends Study (NHATS) showed that the use of everyday technology in older adults was below that of the general population, where only 16% of older adults obtained health information using health technology compared to up to 60% in younger populations [1,10]. However, most NHATS patients considered themselves to be in excellent or very good health. Interestingly, older adults with more comorbidities and those taking more medications were more likely to use digital health technologies in the current study. Our findings differ likely because most of the patients who participated in the WHI have chronic health conditions, unlike prior studies of the older adult population.

Second, disparities in online health information–seeking exist among patients who are ethnic minorities and of a lower socioeconomic status. Consistent with prior studies, our findings add to the evidence that digital health is not reaching all seniors equally [1,3,11-14]. Although recent studies have shown that the gap between those who have access to digital technology and those who do not has become increasingly narrow over time [15], digital health interventions are not reaching a proportion of older patients, and this disparity in access and seeking behavior of online health information may contribute to worsening disparities in health outcomes.

Third, the timing and type of chronic illness may play an important role in the online health information–seeking behavior of older patients. Specifically, patients recently diagnosed with cancer within the last 5 years are more likely to be looking for health information online, even after adjustment for age, suggesting that these patients may have a greater need for digital health resources. This is consistent with findings in the literature. One study has shown that information seeking was reported most frequently by cancer survivors than by the general population [16]. In contrast to previous studies that comprise most of the current literature on the use of digital health technology among older adults, the high proportion of patients with a cancer diagnosis in the WHI provides a unique opportunity to evaluate the use of online health information–seeking among older adults with cancer. Our findings suggest that time since diagnosis of cancer is an important factor in the use of the internet for health among older adults. We also found a relationship between cancer type and online information–seeking behaviors, with patients with colorectal cancer being significantly less likely to use the internet for health information. This mirrors findings from previous studies [16] and may be explained by the fact that the median age at diagnosis of colon cancer in women is 72 years [17], compared with 62 years for women with breast cancer [18].

### Limitations

There are limitations to this study. The WHI participants included only postmenopausal women, thus findings may not be applicable to older men. Further studies are needed to better understand online health information–seeking behaviors in relation to chronic illness among older men. Moreover, the experiences of women in the WHI may not be representative of all older women with chronic health conditions. A prior study has shown that women who participated in the WHI observational study may be healthier than same-age women in the United States [19]. Nevertheless, the diversity of participants’ backgrounds suggests that our findings may be generalizable to a wider population of older women with chronic health conditions. A further limitation is that our findings are based on responses in 2014. Access to and use of digital tools may have become more widespread since participants were surveyed, as a result of continuous technological advancement. Furthermore, we were not able to report the specific reasons why participants did not use the internet to look for health information. It is possible that some women may be interested in looking for health information online but cannot do so due to a lack of access to the internet. It also may be possible that some women benefit from health information available on the internet through their family members or friends [20,21]. Finally, we are only able to report on older adults’ access to and use of the internet to search for health information. We acknowledge that this only reflects one aspect of health information–seeking behavior, and further studies are needed to better understand behaviors such as uses of online portals, online prescribing, and telemedicine.

### Conclusions

Understanding the use of digital health technology among older adults with chronic illnesses and whether they obtain health-related information utilizing the internet are essential pieces of information for designing effective and widespread digital health interventions. Our results show that a significant proportion of older women may not be adequately reached by online information, and thus, there is still a need for more traditional forms of media for the dissemination of health information. This is one of the first studies, to our knowledge, to describe the prevalence of online health information–seeking in older adults with chronic illnesses. Our results provide important information regarding online health information–seeking among older women, particularly those with chronic conditions, and could inform the development of health messaging tailored for this population.

## Appendix

Multimedia Appendix 1 A complete list of technology questions included in the Women’s Health Initiative survey.

## Abbreviations

NHATS: National Health and Aging Trends Study
OR: odds ratio
WHI: Women’s Health Initiative
