# Supplementary material for: Online Health Information–Seeking Among Older Women With Chronic Illness: Analysis of the Women’s Health Initiative
Source: J Med Internet Res. 2020 Apr 9;22(4):e15906. doi: 10.2196/15906 (PMC7319595; doi:10.2196/15906)
Supplement: Multimedia Appendix 1 [file jmir_v22i4e15906_app1.docx]

## Multimedia Appendix 1

1. Do you own a cell phone?

Yes or No

If yes, Do you send or receive text messages on your phone?

Yes or No

1. Do you use a computer (either at home or away from home)?

Yes or No

If yes, Do you use it for email?

No or Yes

Do you use it for Internet?

No or Yes

1. Even if you do not use a computer, do you use a “smart phone,” iPad, or other device for email or the Internet?

No or Yes

1. Do you use the Internet to look for health information?

No or Yes

1. Have you looked at the WHI website (www.whi.org)?

No or Yes
